# Development of Fructooligosaccharide-Rich Sugarcane Juice by Enzymatic Method and Enhancement of Its Microbial Safety Using High-Pressure Processing

**DOI:** 10.3390/foods14193417

**Published:** 2025-10-03

**Authors:** Tanyawat Kaewsalud, Jessica Michelle Liony, Sitthidat Tongdonyod, Suphat Phongthai, Wannaporn Klangpetch

**Affiliations:** 1 Division of Biotechnology, Faculty of Agro-Industry, Chiang Mai University, Chiang Mai 50100, Thailand; tanyawant095@gmail.com; 2 Food Technology Department, Indonesia International Institute for Life Sciences, Jakarta 13210, Indonesia; jessicamichelleliony@gmail.com; 3 Division of Food Science and Technology, Faculty of Agro-Industry, Chiang Mai University, Chiang Mai 50100, Thailand; sitthidat_t@cmu.ac.th (S.T.); suphat.phongthai@cmu.ac.th (S.P.); 4 Cluster of High Value Products from Thai Rice and Plants for Health, Chiang Mai University, Chiang Mai 50100, Thailand

**Keywords:** sugarcane juice, enzymatic treatment, fructooligosaccharides, high-pressure processing

## Abstract

Sugarcane juice (SJ) is a naturally sweet beverage rich in sucrose but prone to microbial contamination, raising concerns among health-conscious consumers. This study aimed to develop a functional SJ enriched with fructooligosaccharides (FOS) using enzymatic treatment, followed by high-pressure processing (HPP) to enhance its safety and quality. The enzymatic conversion of sucrose to FOS was achieved using Pectinex^®^ Ultra SP-L (commercial enzyme), with varying enzyme concentrations, temperatures and incubation times to identify the optimal conditions via response surface methodology (RSM). Under optimal conditions (1000 U/g enzyme concentration, 48 °C, 13 h), sucrose in raw SJ (124.33 g/L) decreased by 59.17 g/L, resulting in maximum reducing sugars (16.02 ± 0.58 g/L) and enhanced FOS yields, notably kestose (2.37 g/L) and nystose (9.35 g/L). After being treated with HPP at 600 MPa for 3 min, *E. coli* K12 and *L. innocua* were effectively inactivated by achieving > 5 log reduction, meeting USFDA standards. Furthermore, it was also observed that HPP could reduce yeast (6.56 × 10^2^ CFU/mL). Meanwhile, mold, *E. coli*, and coliforms were not detected. Additionally, HPP maintained the juice’s physicochemical properties, outperforming thermal pasteurization (85 °C for 10 min) in quality preservation. This study highlights the potential of enzymatic treatment and HPP in improving SJ safety and functionality.

## 1. Introduction

Sugarcane juice (SJ) is a popular beverage extracted from fresh sugarcane stalks, known for its sweet flavor due to a high sucrose content, which constitutes about 15% of the juice [1]. However, the growing concern over excessive sugar consumption has highlighted its link to various chronic diseases, including diabetes, obesity, and cardiovascular diseases [2,3]. As a result, reducing the sucrose content, especially in commonly consumed beverages like SJ, has become crucial for improving public health and preventing these diseases [4].

To address this, enzymatic conversion of sucrose into fructooligosaccharides (FOS) has emerged as an alternative solution. FOS is a type of oligosaccharide with prebiotic properties that help promote the growth of beneficial gut bacteria, contributing to digestive health [5]. Moreover, alternative to sucrose, FOS has the advantage of reducing calorie content by up to 50% and offering only 30% of the sweetness intensity of sucrose [6], making it an attractive sweetener for healthier formulations. β-fructofuranosidase (FFase) is an enzyme that catalyzes hydrolysis of alpha-1,2-glycosidic bonds (glucose and fructose) to form FOS. This enzyme not only releases fructose by breaking the glycosidic bond but also facilitates transfructosylation, transferring fructose to other molecules to form oligosaccharides. The enzymatic conversion of sucrose into FOS is typically achieved through transfructosylation reactions using fructosyltransferase enzymes such as Pectinex^®^ Ultra SP-L [7]. For example, Gonçalves et al. [8] reported that Pectinex^®^ Ultra SP-L at 60 °C for 7 h, pH 5, and a 40:1 substrate-to-enzyme ratio resulted in over 50% FOS production from strawberry juice. In sugarcane syrup, Kamchonemenukool et al. [9] used Pectinex^®^ Ultra SP-L at 50 °C for 4 h, pH 5.2, with a 3.0% enzyme dosage, yielding 63.21% FOS, consisting mainly of 1-kestose and nystose.

While enzymatic methods offer several benefits, including high specificity and milder reaction conditions compared to traditional chemical or physical processes, they still face limitations, particularly in terms of yield. The relatively low production efficiency of FOS through enzymatic treatment necessitates optimization of the process parameters such as enzyme dosage, reaction time, and temperature [10]. In this context, the application of central composite design (CCD), a statistical method within response surface methodology (RSM), provides an effective approach to optimizing these variables. CCD enables a systematic investigation of multiple factors influencing the FOS yield, offering insights into their interactions and allowing fine-tuning of the process for improved output [11].

In addition to improving enzymatic conversion, preserving the quality and safety of FOS-enriched SJ is essential. Due to its high moisture and nutrient content, SJ is prone to microbial contamination, causing short shelf life and nutritional value degradation [12]. Traditional thermal processing methods, especially thermal pasteurization (TP) 85 °C for 10 min, while effective in controlling microbial growth, may compromise the juice’s nutritional and sensory qualities [13]. Therefore, high-pressure processing (HPP) has emerged as a promising non-thermal alternative. By applying high hydrostatic pressure (100–600 MPa), HPP can effectively inactivate microorganisms without the need for heat, thus preserving the nutritional integrity and the FOS content of the juice [14].

Given these advances, the current study aims to explore the development of FOS-rich SJ through optimal enzymatic treatment conditions and the application of HPP. The research focused on evaluating the effect of enzyme optimization on the reduction in sucrose and FOS production, while also assessing the impact of HPP on microbial safety, physicochemical properties and FOS content. This approach provided valuable insights into producing a healthier, safer, and more sustainable beverage product.

## 2. Materials and Methods

### 2.1. Materials and Chemicals

The primary material in this study was fresh SJ obtained from one of the main SJ distributors in Phitsanulok province, Thailand. The commercial enzyme, which is Pectinex^®^ Ultra SP-L (Novozymes, Bagsvaerd, Denmark), 1.0% (*w*/*v*) sucrose solution dissolved in acetate buffer (0.1 M CH_3_COOH, 0,1 M CH_3_COONa, pH 5.5), DNS (3,5-dinitrosalicylic acid) reagent, and distilled water were used when performing enzyme activity assay and enzymatic treatment. Other than that, two surrogate bacteria, namely *Listeria innocua* (JCM 32814) and *Escherichia coli* K12 (JCM 20135), were obtained from the Japan Collection of Microorganisms. Tryptic Soy Agar (TSA, HiMedia, Thane, India), Tryptic Soy Broth (TSB, HiMedia, Thane, India), MacConkey agar (HiMedia, Mumbai, India), Listeria Oxford agar (HiMedia, Mumbai, India), 3M Petrifilm (3M, St. Paul, MN, USA), Potato Dextrose Agar (PDA) (HiMedia, Mumbai, India), Yeast Peptone Dextrose (YPD) agar (HiMedia, Mumbai, India), and saline water 0.85% (*w*/*v*) of sodium chloride solution were prepared for microbial inoculation and microbial analysis steps.

In order to assess the SJ samples, five sugar standards—sucrose (Loba Chemie, Mumbai, India), fructose (Loba Chemie, Mumbai, India), glucose (KemAus, Cherrybrook, NSW, Australia), 1-kestose (ChemFaces Biochemical, Wuhan, China), and nystose (ChemFaces Biochemical, Wuhan, China)—0.5 mg/mL fructose solution, HPLC water, and deionized water were prepared.

### 2.2. SJ Preparation and Quality Assessment

The raw SJ was stored at −18 °C in a freezer until required. Before analysis, the SJ was thawed to room temperature (25 °C). Various physicochemical properties were assessed in this study, including total soluble solids (TSS) using an Atago Master-M hand-held refractometer (ATAGO CO., Ltd., Tokyo, Japan) [15], pH using a FiveGo pH meter F2 (Mettler Toledo, Greifensee, Switzerland) [16], color using a ColorQuest XE spectrophotometer (Hunterlab, Reston, VA, USA) [17], and viscosity using a Brookfield LVDV-II + viscometer (AMETEK, Brookfield, MA, USA) [18]. All analyses were performed in triplicate.

To determine the sugar profile qualitatively and quantitatively, high-performance liquid chromatography (HPLC) was used. The analysis was conducted using the 1260 Infinity II Series HPLC system (Agilent Technologies, Santa Clara, CA, USA), following the procedure outlined by Chumjitchuen et al. [19] with minor adjustments. The HPLC system was equipped with a refractive index detector (RID) (1260 Infinity II, Agilent Technologies, Palo Alto, CA, USA) and a Shodex Asahipak NH2P-50 4E column (Shodex Separation & HPLC, Tokyo, Japan). The mobile phase consisted of a filtered mixture of acetonitrile and HPLC-grade water (70:30 *v*/*v*), and the analysis was performed using isocratic elution at a constant flow rate of 1.00 mL/min. The concentrations of specific sugars in the SJ were calculated by comparing the HPLC results to standard sugar curves.

### 2.3. FOS Production and Optimization

The development of FOS-rich SJ was achieved by converting sucrose to FOS through enzymatic treatment, following the method described by Chumjitchuen et al. [19] with some modifications. Initially, 25 mL of SJ samples were prepared and hydrolyzed using Pectinex^®^ Ultra SP-L at specific concentrations. The incubation was carried out in a shaking water bath (Memmert GmbH + Co. KG, Schwabach, Germany) at 55 °C for 24 h to facilitate the hydrolysis of sucrose into FOS. The samples were immediately placed in ice-cold water after incubation to stop the reaction. The supernatants were collected by centrifugation using an AMT-M04 microcentrifuge (AMTAST, Lakeland, FL, USA) at 6000 rpm (1900× *g*) for 10 min. Finally, the fructose concentration of the samples was determined using the 3,5-dinitrosalicylic acid (DNS) assay [20]. Briefly, the 100 µL sample of the suitable concentration was mixed with 300 µL of DNS solution. The reaction mixture was boiled at 100 °C for 15 min and cooled to room temperature. Following the addition of 600 µL of distilled water, the mixture was vortexed, and its absorbance was measured at 550 nm against a fructose standard.

To optimize the enzymatic treatment conditions for FOS production, response surface methodology (RSM) with central composite design (CCD) was employed. There were three independent factors analyzed using the CCD program, namely enzyme concentration (A), temperature (B), and incubation time (C). Each of these variables was coded and studied at five different levels as shown in Table 1, based on the methodology developed by Kaewsalud et al. [21]. This approach allowed for the investigation of the effects of several independent variables on FOS production, aiming to identify the optimal conditions for generating the highest reducing sugar content in the SJ. The second-order polynomial function derived from the CCD results was used to establish a correlation between the independent variables and the response of generated reducing sugars, as represented in the following equation:

(1) $$Y=\;\beta_{0}+\sum \beta_{1}A\;+\sum \beta_{2}A^{2}+\sum \beta_{3}AB$$

Based on Equation (1), Y was the predicted response of generated reducing sugar (g/L); $\beta_{0}$ was the intercept coefficients; $\beta_{1}$ was the linear coefficients; $\beta_{2}$ was the quadratic coefficients; A and B were the coded independent factors.

### 2.4. High-Pressure Processing and Thermal Pasteurization of SJ

The effect of high-pressure processing (HPP) and thermal pasteurization (TP) on physicochemical properties, sugar content, and microbial inactivation was investigated. To study the microbial inactivation effect, all samples were sterilized before being inoculated with the surrogate microorganisms *E. coli* K12 and *L. innocua*. To prepare the inoculum, both pure cultures were grown in tryptic soy broth (TSB) and incubated at 37 °C for 18–24 h. The cultures were then harvested by centrifugation at 6000 rpm (1900× *g*) and washed three times with a 0.85% (*w*/*v*) NaCl solution. Finally, the bacteria were inoculated into each sample at an approximate initial concentration of 10^6^ CFU/mL before testing.

The HPP treatment followed the research methodology conducted by Kieling et al. [22]. HPP was performed using a high-pressure processing machine (600 MPa/5 L, BaoTou KeFA Co., Ltd., Baotou, Inner Mongolia, China). The samples (30 mL) were placed in polyethylene terephthalate (PET) bottles. The bottles were placed in a cylindrical loading container at an initial temperature of 20 °C and pressurized at 600 MPa for 3 min. Deionized water was used to transmit the pressure. The come-up and depressurization times were approximately 0.5 min and 0.2 min, respectively. Once the HPP process was completed, the samples were immediately cooled, and the HPP-treated products were further analyzed for microbial inactivation. Meanwhile, TP was performed using the pasteurization method according to Mandha et al. [23]. To perform pasteurization, a 200 mL sample was placed in a 250 mL sterile cylinder container. The samples were then heated at 85 °C for 10 min using a stove with constant shaking. The temperature at the center of the glass container was monitored with a sterilized thermometer. Finally, the liquid sample was rapidly cooled and stored for subsequent quality analyses. Moreover, physicochemical properties and sugar content analyses were performed with the unsterile and uninoculated samples.

### 2.5. Analytical Method

#### 2.5.1. Enzymatic Assay

An enzyme activity assay was conducted to observe the ability of Pectinex^®^ Ultra SP-L to perform a transfructosylation reaction by measuring the concentration of reducing sugar released from the breakdown of sucrose molecules. This experiment was done by modifying the method of Gonçalves et al. [24].

Initially, a 1% (*w*/*v*) sucrose solution dissolved in 0.1 M acetate buffer (0.1 M CH_3_COOH, 0.1 M CH_3_COONa, pH 5.5) was prepared to determine the transfructosylation activity of the enzyme. Fifty µL of suitable enzyme concentration (50–100 U/mL) was mixed with 50 µL of sucrose solution. The mixture was incubated in a water bath for 10 min at 55 °C. After incubation, 300 µL of DNS reagent was added to 100 µL of the sample, and the mixture was boiled for 15 min. The sample was then cooled to room temperature and diluted with 600 µL of distilled water. The sample was transferred into a sterile 96-well plate before measuring absorbance at 550 nm using a spectrophotometer (Drawell Scientific, Shanghai, China). The reducing sugar concentration was determined by comparing the sample absorbance value to a fructose standard curve. One unit of enzyme activity (U) was defined as the amount of enzyme that releases 1 µmol per minute under specific assay conditions.

#### 2.5.2. Microbial Analysis

Microbial analysis was conducted to determine the most optimal HPP treatment conditions for reducing the number of *E. coli* K12 and *L. innocua* in the sample by observing the number of colonies present in samples, following the laboratory procedure from Tongdonyod et al. [25]. The enumeration of microbial counts was performed by transferring 20 µL of non-treated, HPP-treated and TP-treated inoculated samples onto MacConkey agar for *E. coli* K12 and Listeria Oxford agar for *L. innocua* strains using the drop plate technique. The plates were then incubated at 37 °C for 20 h. Finally, the number of colonies of each microorganism were counted within the range of 25–250 colonies, and the results were reported as log colony-forming units per mL (log CFU/mL) or further expressed as log reduction.

The methodology from De Souza et al. [26] was used to count the yeast in this study. Each sample was diluted with 0.85% NaCl solution. Then, 100 µL of the sample was spread on YPD agar. The sample was plated in triplicates for each dilution, and the plates were incubated inverted at 30 °C for 2 days.

Meanwhile, mold count was performed following Alam’s method [27]. The spread plate technique was applied, and 100 µL of each sample was spread into PDA in triplicates. The plates were incubated inverted at 30 °C for two days.

For coliform and *E. coli* count, the enumeration was conducted using 3M Petrifilm according to Bird et al. [28]. One milliliter of each sample was pipetted onto 3M Petrifilm in triplicates and spread evenly using a flat object. The Petrifilm was then dried and incubated at 35 °C for 24 h to observe coliform presence, indicated by red colonies with gas bubbles. *E. coli* was observed at 48 h, with blue colonies and gas bubbles.

### 2.6. Statistical Analysis

All experiments were conducted in triplicate to ensure reliable results. Stat-Ease software (Design Expert 6.0.10, Stat-Ease Corporation, Minneapolis, MN, USA) was utilized for the optimization of enzymatic treatment using RSM. The statistical analysis was then performed using IBM SPSS (Statistical Package for Social Sciences) software version 29 (SPSS Inc., Chicago, IL, USA), with a significance threshold set at *p* < 0.05. The independent *t*-test was carried out to analyze significant differences in the sugar content of enzymatic treatment validation samples. Meanwhile, an ANOVA (Analysis of Variance) test was implemented, followed by the Duncan post hoc test to observe significant differences between the samples for other attributes.

## 3. Results

### 3.1. Physicochemical Properties Analysis

Several physicochemical properties of raw SJ were investigated and examined in three replications. As can be seen in Table 2, the TSS value of the raw SJ was 21.5 ± 0.0° Brix. Meanwhile, the pH and viscosity of raw SJ had the approximate values of 5.06 ± 0.03 and 5.25 ± 0.12 cP, respectively. In terms of color attributes, the raw SJ had varying L*, a*, and b* values.

### 3.2. Optimization of Enzymatic Treatments by CCD

The optimal level of three factors, namely enzyme concentration (A), temperature (B) and incubation time (C), and their effects on reducing sugar production were investigated using CCD. The CCD for 17 runs of experimental and corresponding data was shown in Table 3, and the ANOVA result of CCD was presented in Table 4. Moreover, the CCD constructed a quadratic model for reducing sugar, as shown in Equation (2):

Reducing sugar (g/L) = −162.84754 + 0.014136(A) + 6.75620(B) + 0.12273(C) − 6.15346E-006(A^2^) − 0.079977(B^2^) − 0.022336(C^2^) + 6.16330E-004(AB) − 1.77130E-003(AC) + 0.042139(BC) (2)

The program estimating the amount of reducing sugar produced in the experiment had a value ranging from 4.73 to 14.70 g/L based on the regression equation. Furthermore, the result from the actual experiment showed that the highest reducing sugar concentration of 15.72 g/L was obtained from the sample treated for 12 h at 45 °C with an enzyme concentration of 1000 U/g _substrate_ (Table 3).

The enzymatic treatment result was further assessed using CCD analysis. Table 4 presented the ANOVA test result from the program, where the overall quadratic model of the study was found to be significant with the *p*-value of 0.0050. This significant result means that at least one independent factor had a significant or meaningful impact on the reducing sugar production during enzymatic treatment. Meanwhile, the lack of fit value was not significant as the *p*-value result was higher than 0.05, suggesting that the model was fit and adequately describes the relationship between the independent factors and output response.

**Figure 1:**
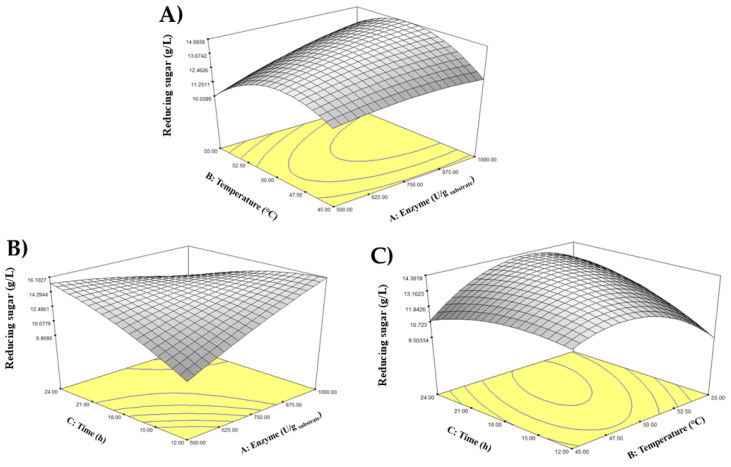
Three-dimensional interactive plots showing the interactions between (**A**) temperature and enzyme concentration, (**B**) incubation time and enzyme concentration, and (**C**) incubation time and temperature.

Futhermore, the CCD program produced a 3D response surface interactive plot to explore the interaction between temperature and enzyme concentration towards reducing sugar production. Referring to Figure 1, it could be seen that the reducing sugar production was slightly increasing when the enzyme concentration increased and became plateau when it reached a higher concentration. On the other hand, the temperature increased the production of reducing sugar up to a certain point and decreased when a higher temperature applied.

The CCD program produced a 3D response surface interactive plot to explore the interaction between temperature and enzyme concentration on reducing sugar production. As shown in Figure 1A, reducing sugar production slightly increased with enzyme concentration and plateaued at higher concentrations. In contrast, temperature initially increased the production of reducing sugar up to a certain point, after which it decreased at higher temperatures. Meanwhile, the interaction between incubation time and enzyme concentration on reducing sugar production is visualized in Figure 1B. A significant interaction occurred between the two independent variables. The highest reducing sugar yield was achieved at the highest enzyme concentration and the longest incubation time. Conversely, the lowest concentration of reducing sugar was produced at the shortest incubation time and lowest enzyme concentration. Moreover, Figure 1C illustrates the interaction between incubation time and temperature on reducing sugar yield, shown as a dome-shaped curve. According to the results, the maximum yield of reducing sugar was achieved when both incubation time and temperature were increased to an optimal moderate point. However, further increases beyond these optimal conditions resulted in a decline in reducing sugar concentration.

After validating the model, the optimal conditions for enzymatic treatment identified through CCD analysis were an enzyme concentration of 978.56 U/g, an incubation temperature of 48.72 °C, and an incubation time of 13.05 h. Under these optimal conditions, the predicted reducing sugar concentration was 15.77 g/L. Experimental validation was conducted with 1000 U/g of enzyme concentration for 13 h at 48 °C. The measured reducing sugar yield was 16.02 ± 0.58 g/L, which was higher than the predicted value, corresponding to a percentage error of 1.58% relative to the predicted value.

The sugar profile and concentration of the validation samples were further analyzed using the HPLC method. The sucrose concentration decreased from 124.33 ± 7.58 g/L (raw SJ) to 65.16 ± 1.16 g/L (FOS-rich SJ), representing an approximate 48% reduction from the initial concentration. Meanwhile, the concentrations of monosaccharide sugars, including fructose and glucose, increased following 13 h of incubation from 6.52 ± 0.72 g/L and 11.86 ± 2.92 g/L to 15.86 ± 0.10 g/L and 28.28 ± 0.19 g/L, respectively. Regarding the FOS products, the amount of kestose (2.37 ± 0.16 g/L) was formed during the incubation process, while the nystose content significantly increased from 4.67 ± 0.88 g/L to 14.02 ± 0.24 g/L.

### 3.3. Effects of HPP on Microbial Inactivation and Physicochemical Properties

FOS-rich SJ samples were inoculated with two surrogate bacteria for microbial inactivation experiments, aimed at identifying the most optimal HPP condition capable of reducing both surrogate bacteria by >5 log reduction. From the study, the FOS-rich SJ samples were processed using HPP at 600 MPa for a holding time of 3 min which represented the mildest condition permitted under the Thai FDA guidelines for HPP pasteurization for low-acid beverages. The results indicated that HPP treatment effectively inactivated *E. coli* K12 and *L. innocua* in the SJ sample, achieving a log reduction of 5.80 ± 0.17 and 5.44 ± 0.16, respectively (Table 5).

Physicochemical properties were analyzed by comparing the HPP-treated SJ with non-treated and TP-treated SJ samples. As shown in Table 6, significant differences were observed in the TSS values across all SJ samples. The highest TSS value was found in the TP-treated SJ (25.3 ± 0.2° Brix), followed by the HPP-treated SJ (23.0 ± 0.0° Brix) and the non-treated SJ (22.5 ± 0.0° Brix). Regarding the pH attribute, the pasteurized sample exhibited a significantly different pH value compared to both the non-treated and HPP-treated SJ. In terms of color, significant differences were observed for the L* and a* values between all three samples, while the b* value showed that the pasteurized sample was significantly different with the non-treated and HPP-treated SJ. Finally, the viscosity of the non-treated SJ was significantly higher (5.96 ± 0.09 cP) compared to both the non-treated and HPP-treated SJ.

For sugar profile, as shown in Table 6, the sucrose concentration in TP-treated SJ (73.08 ± 1.56 g/L) was significantly higher compared to both the HPP-treated and non-treated SJ. Significant differences in fructose and glucose concentrations were observed among all treatments, with the highest fructose and glucose contents found in the TP-treated SJ, at 18.55 ± 0.30 g/L and 39.09 ± 1.16 g/L, respectively. Meanwhile the kestose concentration was represented by the kestose/sucrose ratio. Among the treated samples, HPP-treated SJ showed 0.038 ± 0.00, which was significantly higher than the TP-treated (0.034 ± 0.00). For nystose, as indicated by the nystose/sucrose ratio, ranged from 0.264 ± 0.01 in the non-treated SJ to 0.222 ± 0.01 and 0.246 ± 0.02 in an HPP-treated and TP- treated, respectively. Notably, no significant differences were observed in each treatment.

### 3.4. Microbial Analysis

Microbial analysis was conducted to quantify yeast, mold, *E. coli*, and coliform in the samples and compared with the Thai Community Product Standard (TCPS. 122/2546). As shown in Table 7, the non-treated SJ had the highest yeast content (2.20 × 10^4^ ± 0.61 × 10^4^ CFU/mL), while the HPP-treated SJ had a lower yeast colony count (6.56 × 10^2^ ± 1.26 × 10^2^ CFU/mL), and no yeast was found in the TP-treated SJ. The yeast content in both HPP and TP samples complied with the standard. Additionally, mold, *E. coli*, and coliforms were not detected in any of the samples, indicating that all samples met the regulatory standard.

## 4. Discussion

Thailand stands as a prominent nation in global sugarcane production, yielding a popular, sucrose-rich juice that is widely consumed [29]. However, a significant shift in consumer awareness has highlighted the health implications associated with high sugar intake, linking it to a range of non-communicable chronic diseases such as obesity, diabetes, and cardiovascular conditions [30]. This growing health consciousness has fueled a substantial demand for healthier beverage alternatives with reduced sugar content [31]. To address this challenge, the processing of SJ to mitigate its intrinsic sucrose levels has become an essential area of research and development.

A particularly promising strategy is the enzymatic conversion of sucrose into FOS, which are recognized as valuable prebiotics [32]. FOS not only contributes to gut health by selectively stimulating the growth of beneficial bacteria but also offers the advantage of being low in calories and possessing only about 30% of the sweetness intensity of sucrose, aligning perfectly with current consumer preferences [6]. This study, therefore, focuses on the development of a functional SJ enriched with FOS, achieved through a targeted enzymatic process. Furthermore, to enhance the final product’s safety and preserve its quality, this research employs HPP, a non-thermal preservation technology known for its ability to maintain nutritional and sensory attributes more effectively than traditional thermal pasteurization. The successful integration of these technologies aims to establish a foundational framework for the future development of innovative, health-oriented food products.

The intrinsic physicochemical properties of the raw SJ are critical determinants of the efficiency of the subsequent enzymatic bioconversion process. The SJ utilized in this investigation presented a TSS, indicating a high concentration of sucrose, which serves as an abundant and suitable substrate for FOS synthesis [33]. The pH of the raw SJ was classified as a low-acid food product, a finding consistent with previously reported values around 4.9 [34]. This pH range is advantageous as it aligns well with the optimal operational pH of approximately 5.5 for the selected enzyme, Pectinex^®^ Ultra SP-L. Colorimetric analysis yielded an L* value (lightness), indicating a relatively dark appearance, which is within the reported range of 20.80 to 37.00 for this type of product. The a* and b* values were corresponded to the characteristic greenish-yellow hue of natural SJ [35]. Additionally, the viscosity reflected the high concentration of dissolved sugars contributing to the juice’s body and texture [36].

For the bioconversion of sucrose into FOS, the commercial enzyme preparation Pectinex^®^ Ultra SP-L was selected. This enzyme, derived from the fungus *Aspergillus aculeatus*, is an enzyme complex containing various activities, including pectinase, beta-glucanase, and hemicellulose [37]. Critically for this application, it possesses significant fructosyltransferase (FTase) activity, which is the key catalytic function required to facilitate the transfructosylation reaction for FOS synthesis from sucrose [38]. The efficacy of Pectinex^®^ Ultra SP-L in producing FOS has been well-documented in previous studies using diverse substrates [8,9].

Despite its proven capability, the industrial application of enzymatic processes is often constrained by suboptimal product yields and productivity [39]. To overcome these limitations, process optimization is essential. This study employed a sophisticated statistical methodology, response surface methodology (RSM), to identify the optimal reaction conditions. Specifically, a central composite design (CCD) was utilized, a robust experimental plan well-suited for fitting second-order polynomial models. This design is particularly advantageous as it can accurately model the curvature in response data, an aspect that cannot be captured by simple two-level factorial [40]. The application of RSM coupled with CCD offers significant advantages over traditional one-variable-at-a-time optimization, as it requires fewer experimental runs and, crucially, allows for the evaluation of interaction effects between process variables. The independent variables investigated for optimization in this study were enzyme concentration, reaction temperature, and incubation time, as these are the primary factors governing the efficiency of the enzymatic conversion of sucrose to FOS.

The enzymatic conversion process was evaluated using the total concentration of reducing sugars as the primary response for optimization, as it directly reflects the initial and most crucial step of sucrose conversion. As shown in Table 4, The influence of enzyme concentration on reducing sugar formation reflects hydrolysis. At lower enzyme dosages, the limited number of active sites restricts substrate turnover, whereas increasing enzyme concentration enhances the reaction rate by providing more catalytic sites for sucrose conversion [41]. However, once the active sites outnumber the available substrate molecules, further addition of enzyme does not improve, reducing sugar yield. This plateau is consistent with enzyme kinetics principles, indicating a saturation point where substrate availability becomes the limiting factor [42]. This result aligns with research done by Veljković et al. [43] on sucrose solution, in which raising Pectinex^®^ Ultra SP-L concentration from 1.0% to 2.0% (*v*/*v*) led to a significant increase in the total reducing sugar yield because of the increase in number of enzyme active sites for substrate binding, which accelerated the reaction rate. However, a further increase in enzyme concentration to 5.0% (*v*/*v*) would decrease the reducing sugars yield due to limited substrate availability.

While temperature and incubation time did not emerge as statistically significant primary factors, the positive coefficients in the regression model (Equation (2)) indicated their supportive role in enhancing the yield. Akkarachaneeyakorn et al. [44] mentioned that the enzyme activity was greatly influenced by the environmental condition of enzymatic treatment. A high temperature of enzymatic treatment can speed up the reaction rate of sucrose conversion. However, an extreme temperature beyond the optimal level might also cause the enzyme to denature and lose its activity, resulting in lower yield. This phenomenon could be observed from the curved line shown in Figure 1A and Figure 1C, where the highest reducing sugar yield was achieved at moderate temperature of around 50 °C. The 3D response surface plots illustrated a characteristic parabolic relationship with temperature, with the highest production observed around 48−50 °C, after which thermal denaturation caused a decline in function. Similarly, a longer incubation time improved yield until substrate depletion or product inhibition began to slow the reaction.

Validation of the RSM model confirmed that the optimal conditions were an enzyme concentration of 1000 U/g _substrate_, a temperature of 48 °C, and an incubation time of 13 h. Under these conditions, the actual reducing sugar yield was 16.02 ± 0.58 g/L, closely matching the predicted value and confirming the model’s accuracy. The analysis of the sugar profile revealed a significant 48% reduction in sucrose. This reduction reflects efficient conversion of sucrose into FOS, thereby increasing the yield of prebiotic oligosaccharides. The result was confirmed by the significant increase in both kestose and nystose, consistent with the established sequential mechanism of FOS biosynthesis. This finding aligned with research conducted by Goncalves et al. [8], who found that Pectinex^®^ Ultra SP-L could successfully bioconversion the sucrose to FOS in strawberry preparation from 450 g/L to 83 g/L, with the estimated sucrose conversion of 81.9%.

From a health perspective, the decrease in sucrose content is beneficial, as it lowers the intake of simple sugars while enhancing dietary fiber intake through FOS consumption. Such a profile supports the potential of the treated SJ as a functional food ingredient with prebiotic properties, which may contribute to gut health and overall metabolic benefits [6,32]. In this study, the FOS-rich SJ contained a total FOS concentration of 16.39 g/L, consisting of kestose (2.37 g/L) and nystose (14.02 g/L). Based on a typical serving size of 250 mL, this corresponds to an intake of approximately 4.09 g FOS per day. This level is within the effective range reported to exert prebiotic effects. For instance, Bouhnik et al. [45] demonstrated that a daily FOS intake of 2.5–5.0 g significantly increased *Bifidobacteria*, while Tandon et al. [46] showed that consumption of 2.5–10 g/day promoted the growth of *Bifidobacterium* and *Lactobacillus*. Therefore, the FOS levels obtained in this study might be sufficient to elicit prebiotic activity.

To compare the effects of HPP and TP on the properties of FOS-rich SJ, TP-treated SJ exhibited higher total soluble solids, likely due to water evaporation during heating, which concentrated dissolved solids [47]. Thermal processing also altered color and viscosity, probably resulting from Maillard reactions, degradation of heat-sensitive pigments, and structural changes in juice compounds [48,49]. In contrast, HPP treatment maintained the original color and rheological properties more effectively, indicating that HPP can better preserve the physicochemical quality of FOS-rich SJ while minimizing heat-induced alterations. This observation is consistent with previous studies on SJ and cloudy fruit juices [6,14], which reported that products processed without thermal process maintained their sensory qualities. Thus, the differing impacts of HPP and TP on FOS-rich SJ quality are substantial. HPP demonstrates superior preservation of physicochemical properties and FOS content, whereas TP, despite its microbial inactivation benefits, introduces undesirable changes in color, viscosity, and potentially FOS degradation. A comprehensive understanding of these effects is crucial for guiding the future development of effective and safe functional food products.

Following the enzymatic synthesis, the FOS-rich SJ was processed with HPP to ensure microbial safety. The HPP condition at 600 MPa for 3 min achieved > 5.80 log reduction in *E. coli* K12 and >5.44 log reduction in *L. innocua*, meeting the stringent USFDA safety benchmark of >5 log reduction. HPP induces lethal damage to microbial cells by denaturing critical proteins and disrupting cell membrane integrity, with Gram-negative bacteria like *E. coli* showing slightly higher susceptibility due to their thinner peptidoglycan wall. However, it should be noted that HPP can induce sublethal injury in some bacterial cells, which may not form colonies immediately under standard incubation conditions [50,51]. This phenomenon should be considered when assessing microbial safety.

Microbial analysis is crucial for ensuring product compliance with safety standards. In this study, non-treated SJ exhibited high yeast content, likely due to the development of thermotolerant yeast, which enabled its survival during enzymatic treatment at 48 °C. Conversely, molds, *E. coli*, and coliforms were absent in the non-treated SJ. This suggests that the enzymatic treatment conditions may have created an unfavorable environment for their growth [52]. Experimental results demonstrated significant efficacy of HPP in microbial reduction similar to TP. HPP substantially reduced yeast in SJ from 2.20 × 10^4^ CFU/mL to 6.56 × 10^2^ CFU/mL, an acceptable level according to the product standard. As a non-thermal technology, HPP effectively inactivates various microorganisms, including yeast, mold, and bacteria, in food and beverage products. The mechanism involves applying extremely high pressure, which can damage microbial cell structures, such as cell membranes, cytoplasm, and various organelles. The cell membrane, being highly susceptible to pressure, can lose its function and leak internal components, ultimately leading to cell death [53]. The results are consistent with other studies demonstrating effectiveness of HPP in microbial reduction. For instance, Zhao et al. [54] reported that HPP reduced yeast and mold in cucumber juice by 3 to 4 log CFU/mL. Furthermore, Chai et al. [55] provided evidence that HPP at 550 MPa for 1.5 min could reduce coliforms in Keiskei juice by 6.05 log reduction. Raghubeer et al. [56] also reported that HPP for 3 min under 593 MPa reduced *E. coli* in coconut water to <1 CFU/mL. This study has validated HPP as a superior alternative to TP, not only for its effectiveness in meeting stringent microbial safety standards but also for its ability to better preserve the newly synthesized FOS and the overall physicochemical quality of the juice. Future research should focus on a comprehensive shelf-life study to evaluate FOS stability over time. Additionally, sensory analysis and in vitro studies are essential next steps to confirm consumer acceptance and validate the product’s prebiotic benefits, paving the way for its successful market introduction.

## 5. Conclusions

This research successfully demonstrates the development of a functional, FOS-enriched sugarcane beverage through the integration of optimized enzymatic bioconversion and non-thermal high-pressure processing. The application of RSM with CCD effectively identified the ideal conditions for converting a significant portion of sucrose into FOS. The optimal enzymatic treatment conditions (1000 U/g enzyme concentration, 48 °C, 13 h) resulted in a substantial 48% reduction in sucrose content and a significant increase in FOS compounds, especially kestose (2.37 g/L) and nystose (14.02 g/L). Furthermore, HPP at 600 MPa for 3 min was validated as an effective preservation method, achieving the requisite > 5 log reduction in pathogenic surrogate bacteria (*E. coli* K12 and *L. innocua*) and ensuring the product’s microbial safety in compliance with regulatory standards. Critically, HPP proved superior to conventional thermal pasteurization by better preserving the integrity of the newly synthesized functional FOS, as evidenced by the non-significant change in kestose/sucrose ratio in HPP-treated SJ compared to the significant decrease observed in TP-treated SJ. HPP also maintained the overall physicochemical quality of the FOS-rich SJ (TSS, pH, color, and viscosity) more effectively than TP, which introduced undesirable changes in these attributes due to heat-induced concentration effects and potential degradation. These findings provide a strong basis for producing a value-added, healthier beverage with enhanced safety and maintained nutritional attributes, aligning with the growing consumer demand for reduced-sugar and functional food products. The integrated approach shown in this study offers a promising framework for future innovations in health-oriented food product development.

## Figures and Tables

**Table 1:** Symbol code, range, and levels of three independent factors tested with CCD.

Independent Variable	Symbol	Levels				
		−α	−1	0	+1	+α
Enzyme concentration (U/g _substrate_)	A	250	500	750	1000	1250
Temperature (°C)	B	40	45	50	55	60
Time (h)	C	6	12	18	24	30

**Table 2:** Physicochemical properties of raw SJ.

Attributes	Raw SJ
TSS (° Brix)	21.5 ± 0.0
pH	5.06 ± 0.03
L*	33.11 ± 0.55
a*	−2.76 ± 0.05
b*	7.65 ± 0.07
Viscosity (cP)	5.25 ± 0.12

**Table 3:** CCD result of enzymatic treatment with the predicted and actual values.

Std. Run	Enzyme Concentration (U/g _substrate_)	Temperature (°C)	Incubation Time (h)	Reducing Sugar (g/L)	
				Predicted	Actual
1	500	45	12	9.01	9.21
2	1000	45	12	14.70	15.72
3	500	55	12	4.73	4.90
4	1000	55	12	13.51	12.19
5	500	45	24	12.96	13.44
6	1000	45	24	8.02	7.02
7	500	55	24	13.74	11.88
8	1000	55	24	11.89	10.85
9	250	50	18	10.80	10.89
10	1250	50	18	14.64	15.39
11	750	40	18	6.47	5.70
12	750	60	18	6.05	7.66
13	750	50	6	9.88	9.42
14	750	50	30	12.21	13.50
15	750	50	18	14.26	14.83
16	750	50	18	14.26	15.24
17	750	50	18	14.26	15.24

**Table 4:** ANOVA of variable effects for optimization of reducing sugar production.

Source	Sum of Squares	DF	Mean Square	F-Value	*p*-Value	
Model	175.95	9	19.55	8.54	0.0050 *	significant
A	14.74	1	14.74	6.43	0.0389 *	
B	0.17	1	0.17	0.0752	0.7918	
C	5.43	1	5.43	2.37	0.1675	
A^2^	2.86	1	2.86	2.07	0.3003	
B^2^	77.43	1	77.43	24.66	0.0007 *	
C^2^	12.52	1	12.52	5.58	0.0520	
AB	4.75	1	4.75	1.25	0.1931	
AC	56.48	1	56.48	33.81	0.0016 *	
BC	12.78	1	12.78	5.47	0.0501	
Residual	16.03	7	2.29			
Lack of Fit	14.46	5	2.89	3.67	0.2277	not significant
Pure Error	1.57	2	0.79			
Cor Total	191.98	16				
R-Squared	0.9165					
Adj R-Squared	0.8091					
Pred R-Squared	0.3720					

* Statiscally significant (*p* ≤ 0.05).

**Table 5:** *E. coli* K12 and *L. innocua* inactivation by HPP.

HPP Treatment	Log Reduction (Log CFU/mL)	
600 MPa for 3 min	***E. coli* K12**	***L. innocua***
	5.80 ± 0.17	5.44 ± 0.16

**Table 6:** Physicochemical and sugar profile.

	Raw SJ	Non-Treated SJ	HPP-Treated SJ	TP-Treated SJ
**Physicochemical properties**				
TSS (° Brix)	21.5 ± 0.0 ^d^	22.5 ± 0.0 ^c^	23.0 ± 0.0 ^b^	25.3 ± 0.2 ^a^
pH	5.06 ± 0.03 ^a^	5.00 ± 0.00 ^b^	5.01 ± 0.01 ^b^	5.04 ± 0.01 ^a^
L*	33.11 ± 0.55 ^a^	29.31 ± 0.05 ^d^	29.58 ± 0.09 ^c^	30.91 ± 0.09 ^d^
a*	−2.76 ± 0.05 ^a^	−0.96 ± 0.06 ^c^	−1.06 ± 0.03 ^c^	−1.45 ± 0.03 ^b^
b*	7.65 ± 0.07 ^a^	4.47 ± 0.23 ^c^	4.65 ± 0.05 ^c^	5.62 ± 0.03 ^b^
Viscosity (cP)	5.25 ± 0.12 ^b^	5.32 ± 0.13 ^b^	5.08 ± 0.22 ^b^	5.96 ± 0.09 ^a^
**Sugar concentratio** **n (g/L)**				
Sucrose	124.33 ± 7.58 ^c^	64.25 ± 0.94 ^b^	64.03 ± 1.85 ^b^	73.08 ± 1.56 ^a^
Glucose	11.86 ± 2.92 ^d^	31.04 ± 0.90 ^b^	29.34 ± 0.13 ^c^	39.09 ± 1.16 ^a^
Fructose	6.52 ± 0.72 ^d^	17.07 ± 0.48 ^b^	16.27 ± 0.35 ^c^	18.55 ± 0.30 ^a^
Kestose/Sucrose ratio	-	0.045 ± 0.01 ^a^	0.038 ± 0.00 ^ab^	0.034 ± 0.00 ^b^
Nystose/Sucrose ratio	-	0.264 ± 0.01 ^a^	0.222 ± 0.01 ^a^	0.246 ± 0.02 ^a^

The experiments are performed in triplicate (*n* = 3). The results are reported as mean ± SD. Different letters (a–d) within row are significantly different at *p* < 0.05 according to the analysis by Duncan’s multiple range test.

**Table 7:** Microbial load of non-treated, HPP-treated, and pasteurized SJ.

Microbial Type	Microbial Load (CFU/mL)			Thai Community Product Standard
	Non-Treated SJ	HPP-Treated SJ	TP-Treated SJ	
Yeast	2.20 × 10^4^ ± 0.61 × 10^4^	6.56 × 10^2^ ± 1.26 × 10^2^	ND	**Raw:** <1 × 10^4^ CFU/mL of sample **Pasteurized:** <1 × 10^3^ CFU/mL of sample
Mold	ND	ND	ND	**Raw:** <500 CFU/mL of sample **Pasteurized:** <100 CFU/mL of sample
*E. coli*	ND	ND	ND	**Raw:** <2.2 CFU/100 mL sample **Pasteurized:** Not specified
Coliforms	ND	ND	ND	**Raw:** Not specified **Pasteurized:** <2.2 CFU/100 mL sample

ND = Not detected.

## Data Availability

The data presented in this study are available on request from the corresponding authors.

## Abbreviations

The following abbreviations are used in this manuscript:

ANOVA	Analysis of Variance
CCD	Central Composite Design
DNS	3,4-dinitrosalicylic acid
FOS	Fructooligosaccharides
FFase	β-Fructofuranosidase
FTase	Fructosyltransferase
HPLC	High Performance Liquid Chromatography
HPP	High-Pressure Processing
PDA	Potato Dextrose Agar
PET	Polyethylene Terephthalate
RID	Reactive Index Detectors
RSM	Response Surface Methodology
SJ	Sugarcane Juice
TSS	Total Soluble Solids
YPD	Yeast Peptone Dextrose
